# Impact of community pharmacists on improving polypharmacy in elderly outpatients: a community-based intervention study

**DOI:** 10.1186/s40780-026-00577-z

**Published:** 2026-06-22

**Authors:** Takahiro Furuta, Kazuyoshi Fujitani, Eriko Minami, Yuka Kushida

**Affiliations:** 1 J. MIRAIMEDICAL Co., Ltd. Sakura Pharmacy, 2-12-17 Higashikagaya, Suminoe-Ku, Osaka, 559-0012 Japan; 2 J. MIRAIMEDICAL Co., Ltd. Head Office, Wakita Kyobashi Daiichi Building 8th Floor, 2-5-1, Higashinoda-Machi, Miyakojima-Ku, Osaka, 534-0024 Japan

**Keywords:** Polypharmacy, Community pharmacists, Deprescribing, Elderly outpatients, Medication optimization, Inter-professional collaboration

## Abstract

**Background:**

Polypharmacy, defined as the inappropriate use of multiple concomitant medications, represents a major concern in elderly care and is associated with drug–drug interactions, adverse effects, and poor medication adherence. Previous studies have emphasized the importance of proactive pharmacist involvement in addressing polypharmacy. However, evidence regarding patient outcomes following pharmaceutical interventions remains limited. This study aimed to evaluate whether community pharmacists could contribute to optimizing medication therapy among elderly patients living in the community. Pharmaceutical interventions targeting polypharmacy were implemented with consideration of patient characteristics and caregiver perspectives.

**Methods:**

Questionnaire surveys were conducted among elderly patients receiving six or more daily medications and among caregivers, including helpers and visiting nurses. Cases involving potentially inappropriate prescriptions were identified in elderly outpatients, and deprescribing proposals were communicated to the respective prescribing physicians with patient consent. Follow-up assessments were conducted at least four weeks after intervention in patients whose medications had been deprescribed.

**Results:**

Among elderly respondents, 32.7% expressed a desire to reduce the number of oral medications. Caregivers frequently reported challenges associated with patients receiving multiple prescriptions. Following pharmacist-led interventions, the number of prescribed medications decreased, particularly within the gastrointestinal and pain medication categories. Most patients reported no worsening of symptoms, and some indicated that medication administration had become easier.

**Conclusions:**

These findings suggest that community pharmacists may contribute to improving the quality of medication therapy in elderly outpatients through community-based interventions targeting polypharmacy.

**Graphical Abstract:**

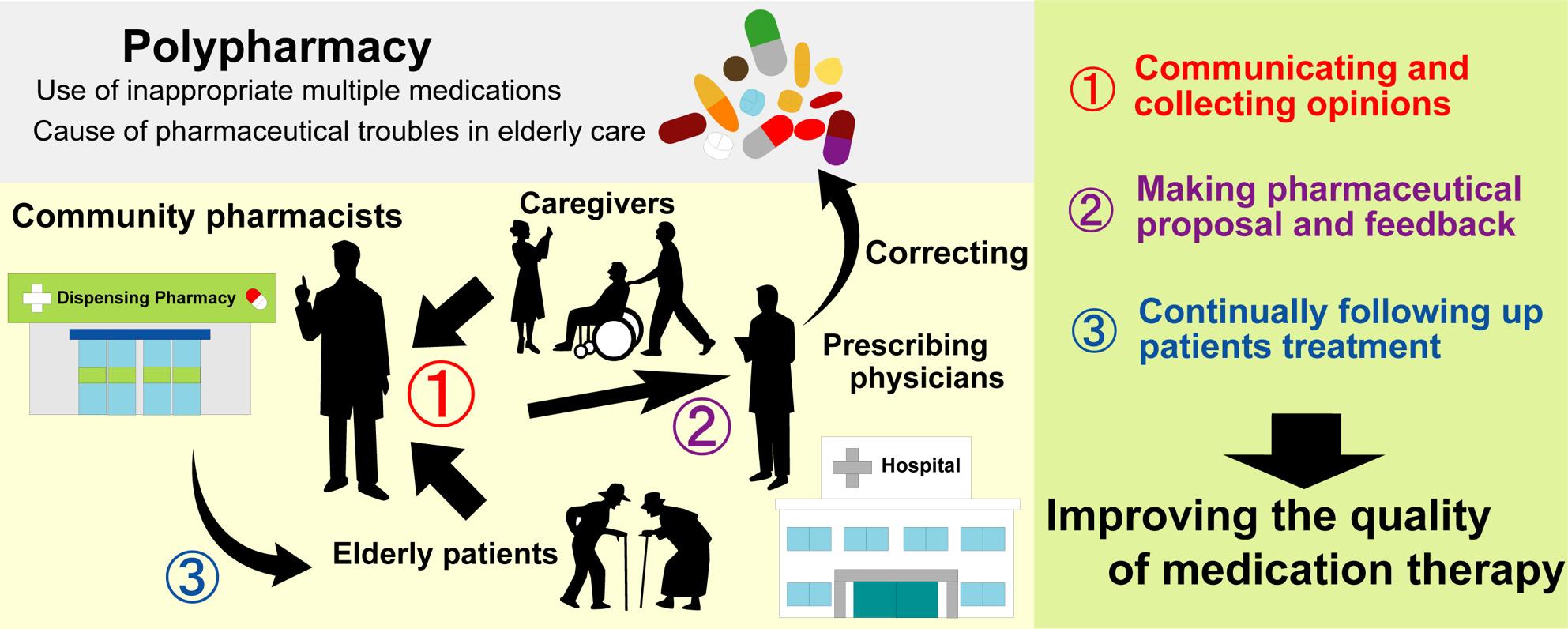

**Supplementary Information:**

The online version contains supplementary material available at 10.1186/s40780-026-00577-z.

## Background

Polypharmacy, the inappropriate use of multiple concomitant medications, is a significant concern in elderly care. It is associated with drug–drug interactions, adverse effects, poor medication adherence, and an increased risk of falls [1, 2]. Although no universally accepted definition based solely on the number of medications exists, previous studies have frequently defined polypharmacy as the regular use of six or more medications [3, 4].

Among the various risk factors for polypharmacy, aging plays a central role. As individuals age, they often develop multiple chronic conditions requiring treatment by different medical specialties [2]. Elderly patients are therefore frequently prescribed numerous medications. This prescribing pattern contributes to increased healthcare costs and represents a significant social concern in Japan. Furthermore, elderly patients in Japan may be more susceptible to adverse events related to polypharmacy than those in other countries [5]. In this context, the development of effective strategies to address and manage polypharmacy is essential.

Hospital pharmacists play an important role in optimizing medication therapy for elderly inpatients by reducing medication-related problems [6, 7]. Various screening tools, including the Beers Criteria and the STOPP/START Criteria, have been used to identify potentially inappropriate prescriptions [6, 8, 9]. Similarly, community pharmacists contribute to the medication management in elderly outpatients by utilizing clinical and contextual information obtained in pharmacy and home-care settings [9, 10]. These findings underscore the importance of proactive pharmacist involvement in addressing polypharmacy in community care.

Many previous investigations have relied on large-scale medical databases or have been confined to single-hospital settings, which may not adequately capture patients’ daily living environments [11, 12]. Consequently, few studies have examined patient-centered outcomes [13], and none have incorporated perspectives from the broader community, such as home helpers, visiting nurses, or family members.

Against this background, the present study aimed to investigate whether community pharmacists can contribute to improving the quality of medication therapy in elderly outpatients by addressing polypharmacy within a community-based framework. Pharmaceutical interventions were implemented with consideration of both patient characteristics and the perspectives of caregivers and other community stakeholders.

## Methods

### Questionnaire survey

Two questionnaire surveys were conducted. The first targeted elderly outpatients (aged ≥ 65 years who were taking ≥ 6 medications) and assessed their current medication therapy. Responses were collected in 2 Sakura pharmacies and 10 Kiraramirai pharmacies (Osaka, Japan) from April 1, 2020 to July 31, 2020. The second survey was administered to caregivers, including helpers and visiting nurses, to evaluate their perspectives on medication-related assistance for elderly patients. Responses were collected in eight care homes and six visiting nursing stations (Osaka, Japan) from November 1, 2023 to March 31, 2024. All responses were categorized according to predefined response options and subsequently analyzed. Prescribed medications were classified into therapeutic categories based on guidelines for the classification of duplicate/concomitant medications [14].

### Pharmaceutical interventions and follow-up of patients

Cases involving potentially inappropriate prescriptions were identified based on reports from outpatients and their caregivers (helpers, nurses, and family members). With the consent of both patients and caregivers, pharmaceutical proposals were communicated to the respective prescribing physicians. Following these interventions, prescribed medications were classified into therapeutic categories according to guidelines [14], and changes in the number of medications prescribed before and after the intervention were calculated (cases: *N* = 33; total number of medications: *n* = 453). The STOPP/START criteria [15] were used as a reference to screen for potentially inappropriate medications. For cases in which the number of prescribed medications was reduced, follow-up assessments were conducted at least 4 weeks after the intervention. Assessments were performed orally by the pharmacist who dispensed the most recent prescriptions and provided medication counseling. Patients’ reported complaints were collected and tallied (*N* = 34).

### Statistical analysis

Statistical analyses were performed using StatView software (version 5.0; Abacus Concepts). Within-patient comparisons before and after intervention were analyzed using the Wilcoxon signed-rank test. A *p*-value < 0.05 was considered statistically significant.

## Results

### Opinions about polypharmacy among elderly outpatients and their caregivers

**Fig. 1:**
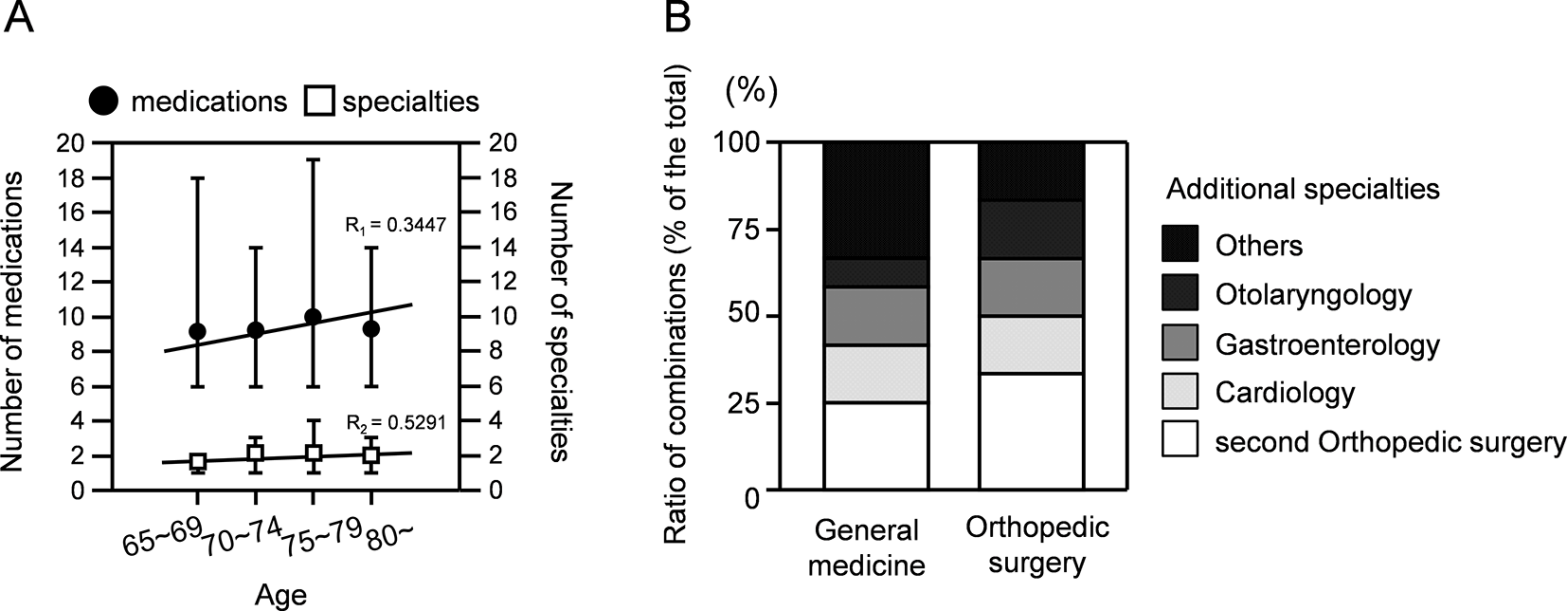
Profiles of elderly outpatients visiting multiple medical specialties and receiving chronic medication therapy. (**A**) Number of prescribed medications (left axis) and number of medical specialties visited (right axis) by age group in elderly outpatients. R indicates correlation coefficients between datasets (R_1_: medications and age; R_2_: specialties and age). Error bars represent maximum and minimum values. (**B**) Combinations of medical specialties prescribing concomitant medications (left bars: in combination with general medicine (*N* = 12); right bars: in combination with orthopedic surgery (*N* = 6)). Panel A: *N* = 116; Panel B: (medications, *n* = 38, respondents, *N* = 18)

Medical information was obtained from elderly outpatients visiting pharmacies, and 116 responses were collected through the questionnaire survey. The number of prescribed medications and the number of medical specialties visited increased with age, demonstrating a positive correlation (correlation coefficients: R_1_ and R_2_ > 0) (Fig. 1A). Among respondents, 93.9% visited a general medicine department and 39.6% visited an orthopedic surgery department. In addition to prescriptions from general medicine or orthopedic surgery departments (*N* = 18), 38 duplicate/concomitant medications were frequently prescribed across different specialties (Fig. 1B).

**Fig. 2:**
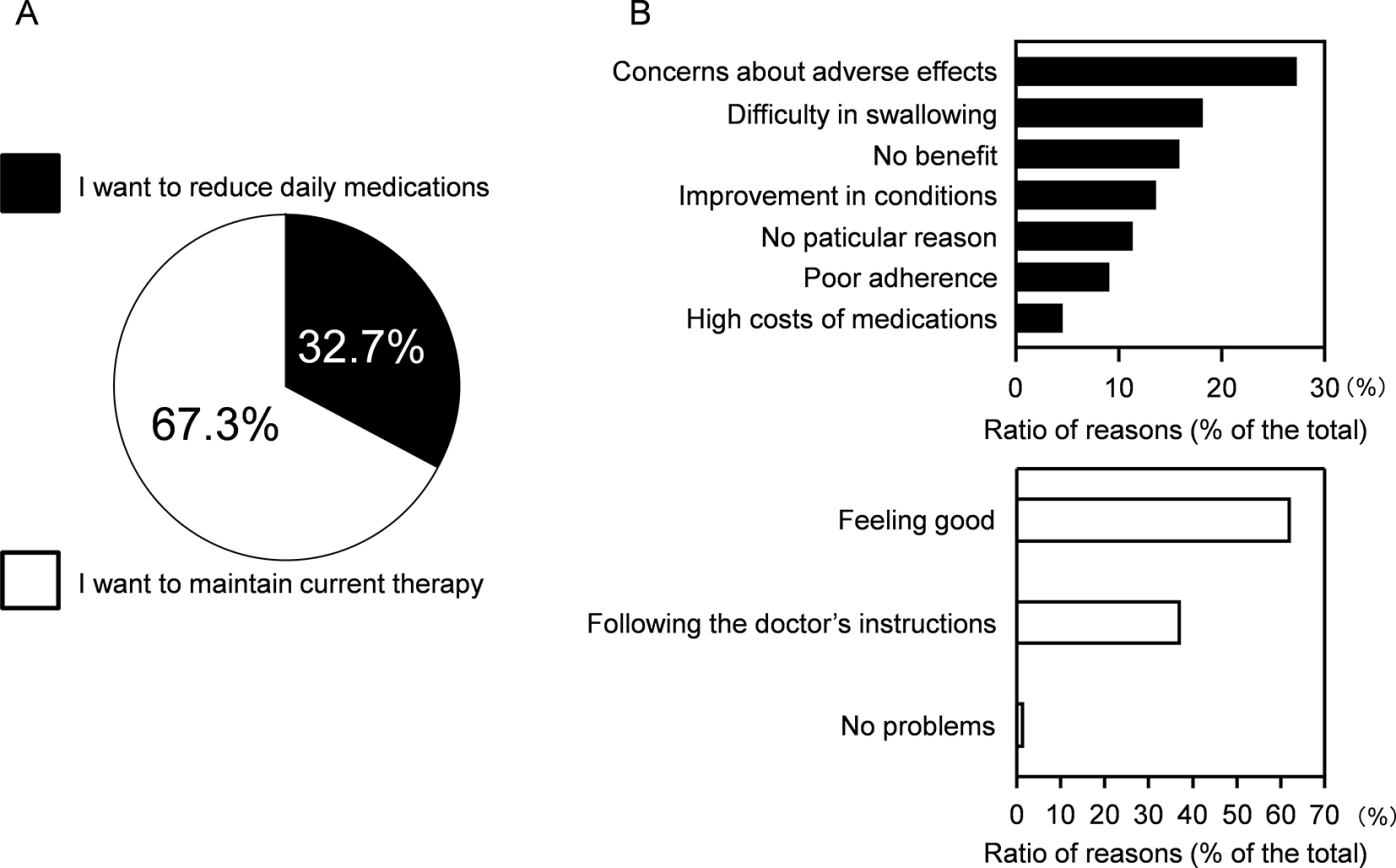
Questionnaire responses of elderly outpatients regarding long-term use of multiple medications. (**A**) Distribution of responses regarding the desire to reduce or maintain current therapy. (**B**) Reasons for each response (black bars: reasons for reduction; white bars: reasons for maintaining therapy). All values represent percentages of total respondents (*N* = 116)

Patients were also surveyed regarding their perceptions of long-term treatment involving multiple medications. A total of 32.7% of respondents expressed a desire to reduce the number of oral medications, whereas the remainder preferred to maintain their current therapy (Fig. 2A). The most frequently reported reason for reducing medications was concern about adverse effects. By contrast, patients who preferred to maintain their current regimen cited reasons such as “I feel good” or “I want to follow the doctor’s instructions” (Fig. 2B).

Caregivers (helpers and nurses) were similarly surveyed, yielding 84 responses (55 from helpers and 29 from nurses). Many responses indicated challenges associated with patients visiting multiple medical institutions and receiving numerous prescribed medications (Table 1).

**Table 1:** Questionnaire responses from caregivers of elderly patients regarding medication-related assistance

I experienced the following situations	Helpers (%)	Nurses (%)
Patients refused to take medications.	54.55	51.72
Elderly patients were prescribed too many daily medications.	32.73	68.97
I was unable to assess the efficacy of medications in patient care.	40.00	51.72
I was unable to assess the necessity of many medications in patient care.	23.64	72.41
I had difficulty determining whether patients’ worsening condition was due to disease progression or adverse drug effects.	40.00	58.62
It was difficult to collaborate with hospitals or clinics.	12.73	20.69
I did not know which physician to consult regarding patients’ worsening conditions.	7.27	24.14
I did not know whom to consult regarding patients’ medical problems.	23.64	31.03
I did not know how to collaborate with community pharmacists.	18.18	55.17

Question statements are presented in the first column. Values represent the proportion (%) of participants who responded “Yes” among total respondents in each group (Helpers: *N* = 55; Nurses: *N* = 29)

### Follow-up investigations for patient outcomes after deprescribing

**Fig. 3:**
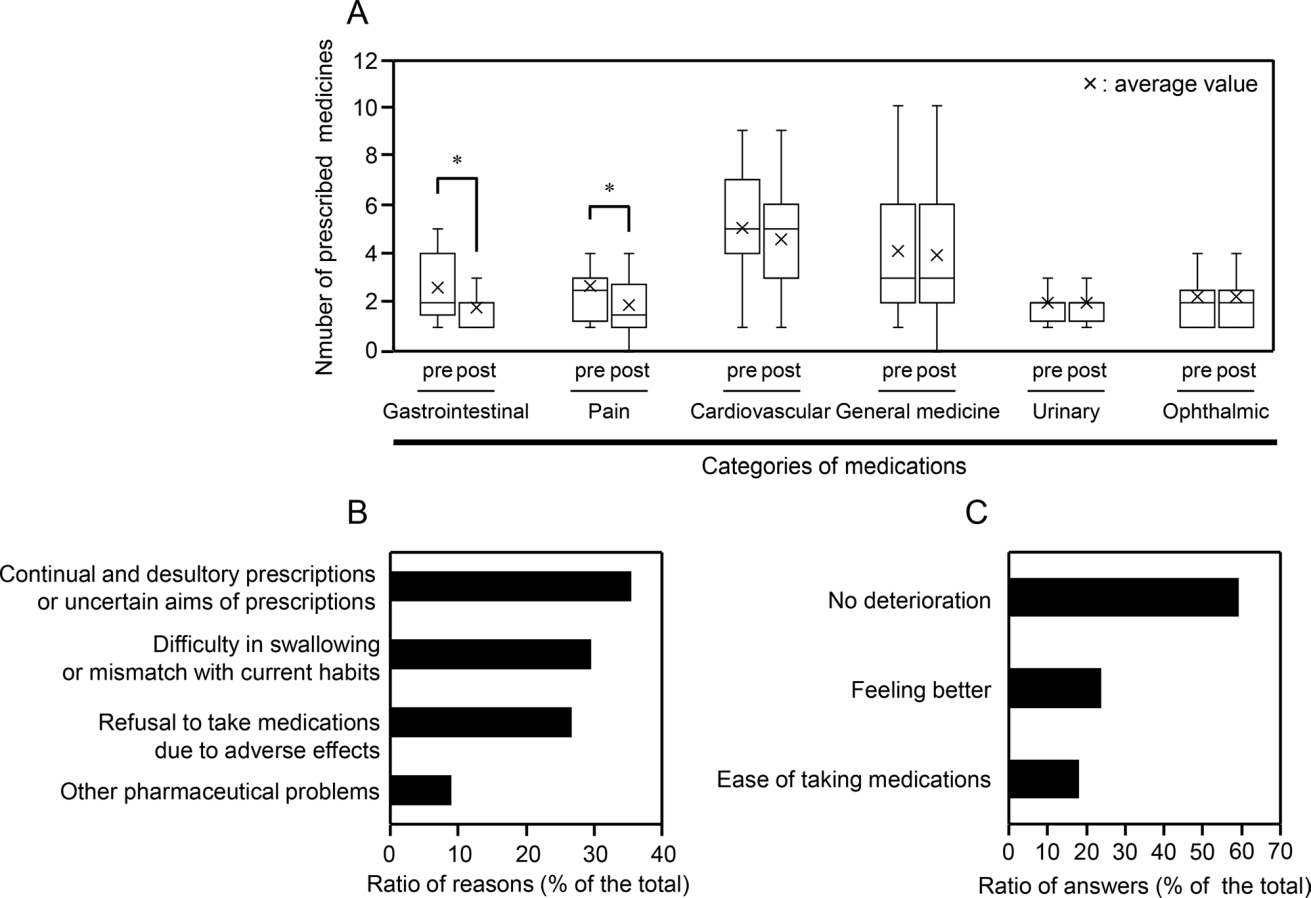
Patient outcomes following pharmacist-led interventions on potentially inappropriate prescriptions. (**A**) Changes in the number of prescribed medications (total medications, *n* = 453) across therapeutic categories pre- and post- intervention. Error bars represent maximum and minimum values. (**B**) Reported reasons for identifying prescriptions as inappropriate. (**C**) Distribution of patient-reported outcomes following deprescribing. Values in panels B and C represent percentages of total cases (*N* = 33). *p < 0.05 vs. pre-intervention

To assess changes following pharmacist intervention, pharmaceutical proposals were communicated to prescribing physicians to address potentially inappropriate prescriptions identified through patient reports. Changes in the number of medications before and after intervention were then evaluated. Outcomes were categorized by medication group (Fig. 3A; Supplementary Table 1). The number of prescribed medications in the gastrointestinal and pain categories decreased significantly compared with pre-intervention levels. By contrast, no statistically significant changes were observed in other categories, including cardiovascular, general medicine, urinary, and ophthalmic medications. The reasons these medications were considered unsuitable for patients’ conditions were further analyzed (Fig. 3B). The most frequently cited reason was prescriptions with unclear or non-specific therapeutic aims. Follow-up assessments were subsequently conducted in patients whose medications had been reduced (Fig. 3C). Most patients reported no deterioration of symptoms, whereas some indicated that medication administration had become easier, and a smaller proportion reported an overall improvement in their condition.

## Discussion

The present study leveraged the position of community pharmacists to obtain perspectives from multiple stakeholders in the community (Fig. 2B; Table 1) and to address potentially inappropriate prescriptions issued by different physicians. The observed reduction in medications suggests that pharmacist-led follow-up may contribute to improving the quality of medication therapy in elderly patients. These findings support the importance of proactive engagement by community pharmacists and highlight the value of close inter-professional collaboration in community-based care.

The magnitude of change differed across medication categories (Fig. 3A). Significant reductions were observed in the gastrointestinal- and pain-related groups, whereas no significant changes were detected in other categories. One possible explanation is that pharmacists were able to act more readily in areas where medication use was closely associated with patients’ subjective complaints or changes in symptoms. By contrast, in conditions such as arteriosclerosis or osteoporosis, where symptoms may be less perceptible, it may have been more difficult to reassess treatment priority and necessity.

Overall, incremental prescribing and limited patient capacity to articulate medication necessity may contribute to polypharmacy. Questionnaire responses indicating a preference to “follow the doctor’s instructions” (Fig. 2B), together with caregiver-reported challenges (Table 1), underscore the need for improved communication among healthcare professionals.

### Study limitations

This study has several limitations to consider when interpreting the results. First, community-level factors may have influenced the findings. Previous research has demonstrated that social determinants can affect polypharmacy, in addition to individual characteristics such as age, body weight, marital status, income, and patient attitudes [12, 16]. As these factors may vary across regions in Japan, the specific characteristics of the study setting may limit generalizability.

Second, the modest sample size limits generalizability. This study focused on individual prescription interventions, which limited the number of cases that could be evaluated within a defined time frame. Broader collaborative studies involving community pharmacists across multiple regions would strengthen the evidence base.

Third, the duration of follow-up was limited. Patient-reported outcomes were assessed at a single time point after deprescribing. Long-term evaluation is necessary to determine the sustained appropriateness of medication changes, particularly as patients’ clinical conditions evolve over time.

Fourth, physician prescribing patterns and pharmacist competencies may have influenced outcomes. Physicians may be cautious about modifying prescriptions initiated by other institutions, and acceptance of pharmacist proposals may vary. Similarly, while community pharmacists are trained in appropriate medication use, variability in addressing polypharmacy may affect intervention implementation. Continued professional development and strengthened inter-professional communication may help mitigate these factors.

## Conclusions

Community pharmacists may contribute to improving the quality of medication therapy for elderly outpatients through community-based interventions targeting polypharmacy. Incorporating patient and caregiver perspectives and strengthening inter-professional collaboration within community healthcare systems may further support efforts to optimize medication use among elderly patients.

## Supplementary Information

Below is the link to the electronic supplementary material.

Supplementary Material 1

## Data Availability

No datasets were generated or analyzed during the current study.
