# Supplementary material for: Impact of community pharmacists on improving polypharmacy in elderly outpatients: a community-based intervention study
Source: J Pharm Health Care Sci. 2026 Jun 22;12:59. doi: 10.1186/s40780-026-00577-z (PMC13285662; doi:10.1186/s40780-026-00577-z)
Supplement: Supplementary file 1 — Supplementary Material 1 [file 40780_2026_577_MOESM1_ESM.doc]

| Medication groups | -intervention | Min | 1st Quartile | Median | 3rd Quartile | Max | Inter Quartile Range | Mean | 95%CI |
| --- | --- | --- | --- | --- | --- | --- | --- | --- | --- |
| Gastrointestinal | Pre | 1.00 | 1.50 | 2.00 | 4.00 | 5.00 | 2.50 | 2.61 | 2.05～3.17 |
|  | Post | 1.00 | 1.00 | 1.00 | 2.00 | 3.00 | 1.00 | 1.80 | 1.41～2.19 |
| Pain | Pre | 1.00 | 1.25 | 2.50 | 3.00 | 4.00 | 1.75 | 2.68 | 1.97～3.39 |
|  | Post | 0 | 1.00 | 1.50 | 2.75 | 4.00 | 1.75 | 1.90 | 1.34～2.46 |
| Cardiovascular | Pre | 1.00 | 4.00 | 5.00 | 7.00 | 9.00 | 3.00 | 5.03 | 4.31～5.74 |
|  | Post | 1.00 | 3.00 | 5.00 | 6.00 | 9.00 | 3.00 | 4.58 | 3.86～5.30 |
| General medicine | Pre | 1.00 | 2.00 | 3.00 | 6.00 | 10.0 | 4.00 | 4.10 | 2.81～5.39 |
|  | Post | 0 | 2.00 | 3.00 | 6.00 | 10.0 | 4.00 | 3.93 | 2.62～5.23 |
| Urinary | Pre | 1.00 | 1.25 | 2.00 | 2.00 | 3.00 | 0.75 | 2.00 | 1.41～2.58 |
|  | Post | 1.00 | 1.25 | 2.00 | 2.00 | 3.00 | 0.75 | 2.00 | 1.41～2.58 |
| Ophthalmic | Pre | 1.00 | 1.00 | 2.00 | 2.5 | 4.00 | 1.50 | 2.25 | 1.21～3.28 |
|  | Post | 1.00 | 1.00 | 2.00 | 2.5 | 4.00 | 1.50 | 2.25 | 1.21～3.28 |

**Supplementary Table 1 Detailed number of prescribed medications before and after the intervention.**
